# Supplementary material for: Exploring the Antibacterial and Antibiofilm Efficacy of Silver Nanoparticles Biosynthesized Using Punica granatum Leaves
Source: Molecules. 2021 Sep 23;26(19):5762. doi: 10.3390/molecules26195762 (PMC8510064; doi:10.3390/molecules26195762)
Supplement: Supplementary file 1 [file molecules-26-05762-s001.zip › molecules-1357401-supplementary.pptx]

## Slide 1
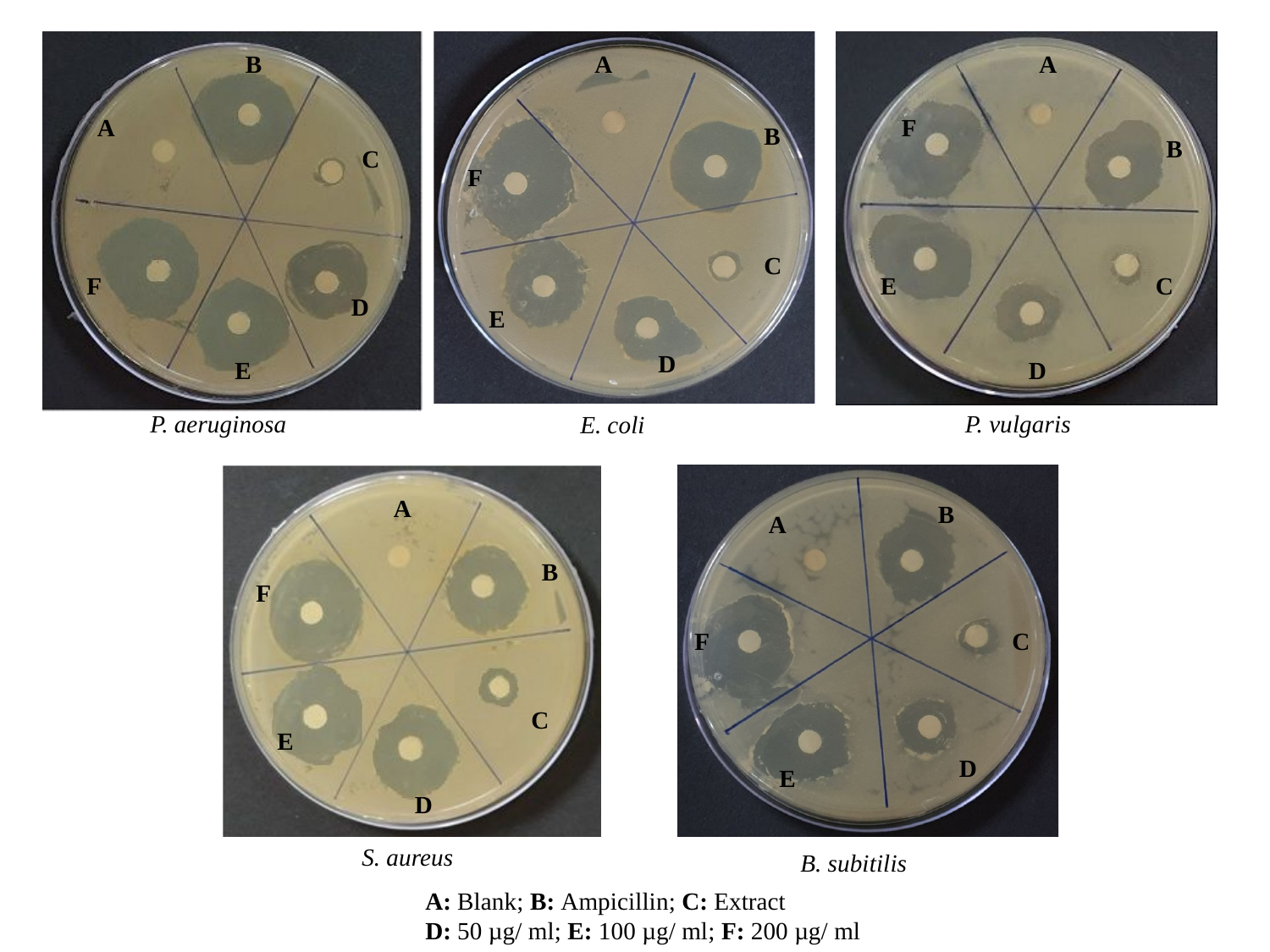

B
A
C
F
D
E
P. aeruginosa
A
B
F
C
E
D
E. coli
A
F
B
E
C
D
P. vulgaris
A
B
F
C
E
D
S. aureus
B
A
F
C
D
E
B. subitilis
A: Blank; B: Ampicillin; C: Extract
D: 50 µg/ ml; E: 100 µg/ ml; F: 200 µg/ ml
